# Magnetic resonance imaging for lung cancer detection: Experience in a population of more than 10,000 healthy individuals

**DOI:** 10.1186/1471-2407-11-242

**Published:** 2011-06-13

**Authors:** Nai-Yuan Wu, Hui-Cheng Cheng, James S Ko, Yu-Chen Cheng, Po-Wei Lin, Wei-Chan Lin, Cheng-Yen Chang, Der-Ming Liou

**Affiliations:** 1 VGH-HT Imaging Center, Taipei Veterans General Hospital, Taipei, Taiwan; 2 Institute of BioMedical Informatics, National Yang-Ming University, Taipei, Taiwan; 3 Department of Family Medicine, Taipei Veterans General Hospital, Taipei, Taiwan; 4 Department of Radiology, Taipei Veterans General Hospital, Taipei, Taiwan

## Abstract

**Background:**

Recent refinements of lung MRI techniques have reduced the examination time and improved diagnostic sensitivity and specificity. We conducted a study to assess the feasibility of MRI for the detection of primary lung cancer in asymptomatic individuals.

**Methods:**

A retrospective chart review was performed on images of lung parenchyma, which were extracted from whole-body MRI examinations between October 2000 and December 2007. 11,766 consecutive healthy individuals (mean age, 50.4 years; 56.8% male) were scanned using one of two 1.5-T scanners (Sonata and Sonata Maestro, Siemens Medical Solutions, Erlangen, Germany). The standard protocol included a quick whole-lung survey with T2-weighted 2-dimensional half Fourier acquisition single shot turbo spin echo (HASTE) and 3-dimensional volumetric interpolated breath-hold examination (VIBE). Total examination time was less than 10 minutes, and scanning time was only 5 minutes. Prompt referrals and follow-ups were arranged in cases of suspicious lung nodules.

**Results:**

A total of 559 individuals (4.8%) had suspicious lung nodules. A total of 49 primary lung cancers were diagnosed in 46 individuals: 41 prevalence cancers and 8 incidence cancers. The overall detection rate of primary lung cancers was 0.4%. For smokers aged 51 to 70 years, the detection rate was 1.4%. TNM stage I disease accounted for 37 (75.5%). The mean size of detected lung cancers was 1.98 cm (median, 1.5 cm; range, 0.5-8.2 cm). The most histological types were adenocarcinoma in 38 (77.6%).

**Conclusion:**

Rapid zero-dose MRI can be used for lung cancer detection in a healthy population.

## Background

Lung cancer is the leading cause of cancer death worldwide [1-3]. The overall 5-year survival rate is approximately 15% in the United States and less than 10% in Europe [4-6]. Because most lung cancers generate no symptoms at early stages, they are usually diagnosed at an advanced stage and have a poor prognosis. However, many lung cancer deaths could be avoided if tumors were detected at an early stage when they were still resectable. For stage Ia non-small cell lung cancer, the 5-year survival rate is higher than 80%. Prognosis and treatment outcomes have been found to be related to the disease stage at the time of diagnosis [7-9]. Various modalities have been investigated for detecting early lung cancer and consequently reducing lung cancer mortality. In the 1970s, several randomized controlled trials of chest radiography, alone or combined with sputum cytology, were performed. However, because of the poor sensitivity of these methods, the reported data indicated no evidence of benefit in terms of reduction in lung cancer mortality [10-12]. In the 1990s, many observational clinical trials were begun of low-dose spiral computed tomography (LDCT). Those studies showed that although LDCT can detect more early lung cancers than chest radiography, it does not result in a decrease in lung cancer deaths [13-19].

Until recently, magnetic resonance imaging (MRI) was regarded as an inappropriate tool for lung cancer screening because of its insufficient anatomic detail, along with being time-consuming and expensive. The advanced MRI techniques for lungs have reduced the examination time to less than 10 minute (< 8 breath-holds) and improved diagnostic sensitivity and specificity. We conducted a study to assess the feasibility of performing rapid MRI for lung cancer detection. We report our techniques and findings herein.

## Methods

### Population

In October 2000, Taipei Veterans General Hospital in Taiwan launched whole-body MRI examinations in routine clinical practice [20]. All examinations were paid for by the examinees, who were referred from clinician or self-registered. So far, more than 20,000 examinations have been carried out for 17,000 people. We performed a retrospective chart review for all whole-body MRI examinations between October 2000 and December 2007. Institutional Review Board of Taipei Veterans General Hospital in Taiwan approved this retrospective chart review and waived the requirement for informed consent because all images and follow-up data were obtained as part of routine clinical care (VGHIRB No: 201006010IC).

For this study, analysis was focused on images of lung parenchyma. We did not take into account imaging findings of the mediastinum, chest wall, diaphragm, or metastatic lung lesions diagnosed simultaneously with primary cancer. Examinees with a prior history of cancer were excluded from the study. Examinees with normal lung imaging but diagnosed with other cancers by whole-body MRI were also excluded. Demographic details of examinees as well as smoking habits and prior medical history were analyzed.

### MRI

**Figure 1:**
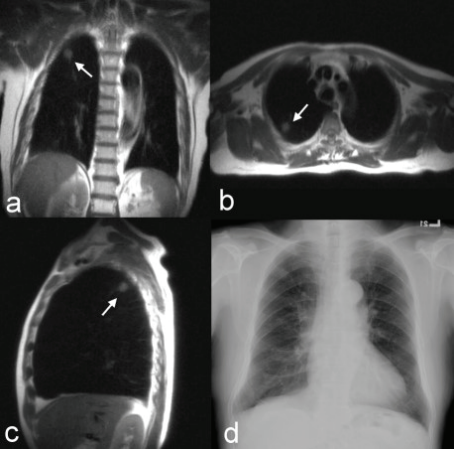
**The HASTE images from MRI**. The (a) coronal, (b) axial, and (c) sagittal images showed an irregular consolidated mass about 1.2 cm in diameter at the posterior segment of the right upper lobe. Under black blood preparation, the lesion easily stood out from the clear background without the appearance of any vessels. (d) The findings from chest radiography were negative. The nodule was surgically proved to be squamous cell carcinoma, stage Ia.

All images were acquired using either one of two 1.5-T scanners (Sonata and Sonata Maestro, Siemens Medical Solutions, Erlangen, Germany). The standard protocols followed for lung examinations consisted of two components. The first involves a quick whole-lung survey with T2-weighted 2-dimensional half Fourier acquisition single shot turbo spin echo (2D HASTE), using the turbo spin echo technique with double inversion recovery black blood preparation electrocardiogram triggered in axial, coronal, and sagittal orientations with repetition time (TR) of one RR interval, echo time (TE) of 41 ms, flip angle of 160°, slice thickness of 6 mm, and matrix of 172 × 256. The examination time was less than 3 minutes (Figure 1).

**Figure 2:**
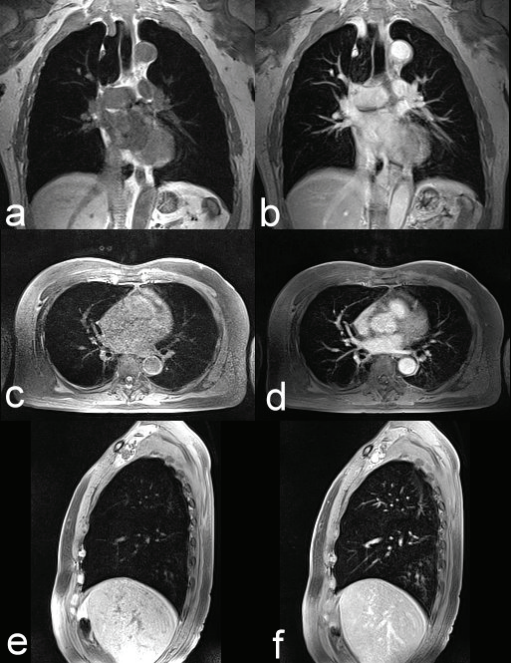
**Comparison of VIBE images from MRI without and with the use of contrast**. The (a) noncontrasted coronal, (b) contrasted coronal, (c) noncontrasted axial, (d) contrasted axial, (e) noncontrasted sagittal, and (f) contrasted sagittal VIBE images can display clear branches of pulmonary vessels and bronchial tree with minimal pulsation artifacts in normal lung parenchyma.

The second protocol component was performed with 3-dimensional axial image acquisition using the volumetric interpolated breath-hold examination (3D VIBE) technique with fat suppression, TR/TE of 4.9 ms/2.1 ms, flip angle of 12°, field of view (FOV) of 320-360 mm, matrix of 176 × 256, and slice thickness of 3 mm with 64 slices per breath hold. The duration time for this sequence was less than 3 minutes (Figure 2).

All examinees could choose their own MRI examinations, either noncontrasted whole-body MRI or with additional contrasted MRI examinations, such as contrast magnetic resonance angiography (MRA) or breast MRI. For examinees who had additional contrasted MRI, HASTE was applied before contrast agent injection, and VIBE was applied after contrast agent injection using 0.5 mmol/kg of gadodiamide (Omniscan) at an injection rate of 3 mL/sec.

### Image Analysis

All images were viewed by 1 of 5 radiologists (HCC, JSK, YCC, PWL, WCL), who had 4 to 19 years of experience (average, 9.6 years) after board certification. We classified and defined all lesions as (1) normal or extrapulmonary abnormality: negative result in lungs or lesions in the extrapulmonary area, for instance, in the neck or abdomen, (2) lung abnormality of little clinical significance: pulmonary lesions rather than nodules, for instance, pleural thickening or fibrosis, (3) probable benignancy: lung nodules measuring ≤ 0.5 cm with well-defined border and visible only by VIBE and not by HASTE, (4) indeterminate nodules: lung nodules measuring ≤ 0.5 cm with mild irregular border and visible only by VIBE and not by HASTE, (5) possible malignancy: lung nodules measuring > 0.5 cm and <1 cm and visible only by VIBE and not by HASTE, (6) probable malignancy: lung nodules visible by both VIBE and HASTE; or lung nodules measuring ≥ 1 cm; or newly developed lung nodules at the second or more MRI examinations. The latter three were considered to be suspicious lung nodules [16,20].

For examinees with possible malignancies or indeterminate small lung nodules, serial follow-ups were arranged either by MRI or by standard-dose computed tomography (CT) every 3 to 6 months for a minimum of 24 months. Nodules remaining the same size or regressing during follow-up for at least 2 years were considered to be benign [21,22]. When growth was found, referrals to thoracic surgeons were arranged. For examinees with probable malignancies, immediate standard-dose CT and referrals to thoracic surgeons were arranged. The decision as to how to perform investigative procedures was left to the referred surgeon. All cytological and histological findings from biopsies and surgical specimens were documented. When a cancer was diagnosed, the examinee received standard care, including tumor staging and appropriate treatment. Outcomes of referrals were followed, and cancer-related details were logged.

### Statistical analysis

Statistical analysis was performed using Microsoft Excel 2003 (Microsoft Corporation, Redmond, WA, USA). The results are presented as mean, median, and range. The Yates-corrected chi-Square test was used for categorical variables to evaluate difference. Results were considered statistically significant at *p *< 0.05.

## Results

From October 2000 to December 2007, a total of 14,040 lung MRI examinations were performed in 11,766 consecutive individuals, who were asymptomatic and had no prior cancer history in the lung or elsewhere. The mean age was 50.4 years (range, 11-94 years), and 56.8% of examinees were male. Never-smokers accounted for 89.2% and smokers for 10.8%. The median number of pack-years of smoking was 22.5 (range, 2-90). No patients had a history of asbestos exposure. Characteristics of the examinees are shown in Table 1. Of the 11,766 examinees, 10,160 (86.3%) underwent lung MRI once (only the prevalence examination), 1172 (10%) twice, and 434 (3.7%) three times or more. Of the 14,040 lung MRI studies, 5037 were performed without the use of a contrast agent and 9003 with a contrast agent. Table 2 shows the interindividual comparison of lung MRI examinations. Contrasted MRI disclosed significantly more lung nodules, both benign and suspicious, than noncontrasted MRI (*p *< 0.001).

**Table 1:** Characteristics of individuals undergoing lung magnetic resonance imaging examination

Age group	Never-smoker (female/male)		Smoker (female/male)	
11-20	34	(14/20)	0	(0/0)
21-30	253	(144/109)	41	(7/34)
31-40	1407	(743/664)	219	(28/191)
41-50	3845	(1807/2038)	538	(41/497)
51-60	2869	(1306/1563)	341	(27/314)
61-70	1394	(689/705)	93	(3/90)
71-80	598	(249/349)	29	(4/25)
81-90	96	(22/74)	5	(0/5)
91-100	4	(1/3)	0	(0/0)
				
Total	10,500	(4975/5525)	1266	(110/1156)

**Table 2:** Interindividual comparison of noncontrasted and contrasted lung magnetic resonance imaginga

Result	Non contrasted		Contrasted		Total	
Normal or extrapulmonary abnormality	4096	(81.3)	6936	(77.0)	11,032	(78.6)
Lung abnormality of little clinical significance	684	(13.6)	1453	(16.1)	2137	(15.2)
Probable benignancy	88	(1.7)	214	(2.4)	302	(2.2)
Possible malignancy	81	(1.6)	183	(2.0)	264	(1.9)
Probable malignancy	23	(0.5)	56	(0.6)	79	(0.6)
Indeterminate small nodule measuring ≤0.5 cm	65	(1.3)	161	(1.8)	226	(1.6)
						
Subtotal of suspicious lung nodules^b^	169	(3.4)	400	(4.4)	569	(4.1)
Total	5037	(100)	9003	(100)	14,040	(100)

Note: Data are given as number of examinations. Numbers in parentheses are percentages.

^a^There was a significant difference between noncontrasted MRI and contrasted MRI by Yates-corrected chi-square test (*p *< 0.001).

^b^Possible malignancy, probable malignancy, and indeterminate small nodule are considered to be suspicious lung nodules.

A total of 559 people (4.8%) were found to have suspicious lung nodules. Of the 559, 376 (67.3%) had nodules considered to be benign during follow-up and 138 (24.7%) were lost to follow-up. A total of 49 primary lung cancers were diagnosed in 46 examinees: 41 prevalence cancers and 8 incidence cancers. The rate of primary lung cancers detected by MRI was 0.4% (49 of 11,766). The detection rate was 0.9% (11 of 1266) for smokers, and 0.4% (38 of 10,500) for never-smokers (Table 3). There was no significant difference of cancer detection rate between smokers and never-smokers (*p *= 0.11).

**Table 3:** Distribution of detected primary lung cancers by magnetic resonance imaging

	Never-smoker				Smoker					
	
Age group	Female		Male		Female		Male		Total	
31-40	1	(0.1)	0	(0)	0	(0)	1	(0.5)	2	(0.1)
41-50	4	(0.2)	2	(0.1)	1	(2.4)	1	(0.2)	8	(0.2)
51-60	3	(0.2)	7	(0.4)	0	(0)	5	(1.6)	15	(0.5)
61-70	10	(1.5)	3	(0.4)	0	(0)	1	(1.1)	14	(0.9)
71-80	4	(1.6)	2	(0.6)	0	(0)	2	(8.0)	8	(1.3)
81-90	1	(4.5)	1	(1.4)	0	NA^b^	0	(0)	2	(2.0)
										
Total^a^	23	(0.5)	15	(0.3)	1	(0.9)	10	(0.9)	49	(0.4)

Note: Data are given as number of lung cancers. Numbers in parentheses are percentages.

^a^There was no significant difference of cancer detection rate between smokers and never-smokers by Yates-corrected chi-square test (*p *= 0.11).

^b^Not applicable.

Of the 49 lung cancers, the mean age of the individual at diagnosis was 60.8 years (range, 36-87 years), and 25 (51.0%) were in males. Eleven cases were in people who had smoked, and the median number of pack-years of smoking was 35 (range, 10-80). Characteristics of individuals with detected primary lung cancers are shown in Table 3. Suspected lung cancers were subjected to various procedures for proper histopathological documentation. Thoracotomy (including video-assisted thoracoscopic surgery) was performed for 40 cancers, lobectomy for 33 cancers, wedge resection for 6, and segmentectomy for 1. Three cancers were histologically proved by bronchoscopic biopsy, 1 by CT-guided lung biopsy, 1 by sputum cytology, 1 by biopsy at supraclavicular lymph node, and 1 by endoscopic biopsy at metastatic duodenal lesion. Two individuals refused any procedures or therapies and later developed rib metastases or progressive pulmonary nodules. Forty cancers had pathological staging, and 9 had clinical staging. Twenty-five cancers were TNM stage Ia disease (51.0%), 12 stage Ib disease (24.5%), 4 stage IIIa (8.2%), 2 stage IIIb (4.1%), and 6 stage IV disease (12.2%). The mean size of detected lung cancers was 1.98 cm (median, 1.5 cm; range, 0.5-8.2 cm). Thirty-six cancers (73.5%) had a primary tumor measuring ≤3 cm, and 29 (59.2%) ≤2 cm. Histological types were as follows: 38 adenocarcinoma (77.6%), 5 bronchioloalveolar carcinoma (10.2%), 2 large cell carcinoma (4.1%), 1 squamous cell carcinoma (2.0%), and 1 carcinoid tumorlet (2.0%). There were 3 female individuals with synchronous double primary lung cancers. One biopsy proved to be non-small cell lung carcinoma with no primary tumor origin in the lungs when retrospectively and prospectively assessed. Two cases did not have tissue proof because the individuals refused any procedures.

Of the 8 incidence cancers, 5 were diagnosed at the second MRI examination, with a mean interval of 3.3 years (range, 2-5.5 years). Two cancers were diagnosed at the third annual MRI examination. The comparison of prevalence cancers and incidence cancers is shown in Table 4. The mean size of the incidence cancers was 1.8 cm, compared with 2.0 cm for the prevalence cancers.

**Table 4:** Comparison between prevalence lung cancers and incidence lung cancers by magnetic resonance imaging examination

	Prevalence cancer	Incidence cancer
Mean size (cm)	2.0	1.8
		
Cancer detection rate (%)		
Smoker		
Male	0.7	0.9
Female	0.9	NA^a^
Never-smoker		
Male	0.2	0.7
Female	0.4	0.2

^a^Not applicable.

Follow-up (duration, 2-9 years) for the 49 cancers showed 8 deaths due to lung cancer, 2 stage IIIa cancers, 2 stage IIIb cancers, and 4 stage IV cancers. One stage Ib individual developed recurrent tumors 2 years later and underwent pulmonary resection again. Thirty-three individuals (67.3%) were alive and free of disease at the time of this writing.

Eight false-positive individuals whose standard-dose CT also suggested malignancies underwent invasive procedures: 2 CT-guided biopsies and 6 thoracotomies (including video-assisted thoracoscopic surgery). Infectious diseases were histologically confirmed in these 8 people, with cryptococcal infections in 6.

## Discussion

To date, several studies have investigated the feasibility of LDCT for screening for primary lung cancers. By enrolling heavy smokers and older people, these studies targeted groups at high risk for lung cancer, resulting in cancer detection rates ranging from 0.4% to 2.7% and lung nodule prevalence rates ranging from 5.1% to 43% [13-19]. In our study, there were no limitations on smoking history and age because all examinations were self-paid and all examinees were self-enrolled. Consequently, our group included a higher proportion of never-smokers and a wider age range (11-94 years). If we calculate the results for smokers between 51 and 70 years old, as did the LDCT studies, the detection rate is 1.4% (6 of 434), and the prevalence of suspicious lung nodules is 9.0% (39 of 434). The proportion of lung cancers among suspicious nodules detected by MRI was 15.4% (6 of 39), higher than in reports for LDCT, which range from 2.2% to 11.6%. While using standard-dose CT as a standard-of reference for nodule detection, previous studies have showed that MRI can achieve high sensitivity for nodules larger than 1 cm, which are clinical approachable [23,24]. Undoubtedly LDCT is more sensitive than MRI in detecting lung nodules and includes more and smaller nodules. However, for any diagnostic tool, higher sensitivity is usually accompanied by lower specificity. Most of the additional lung nodules detected by LDCT are smaller than 0.5 cm. According to guidelines for managing small lung nodules, nodules smaller than 1 cm are associated with more follow-up visits but not more lung cancers [21,22]. We believe that it is why our results showed that MRI had a lower prevalence of suspicious nodules than did LDCT but had a cancer detection rate similar to that of LDCT.

Contrasted MRI can detect more morphological details than noncontrasted MRI, and is especially better at depicting peripheral one-third parenchymal vascular markings (around 0.2 cm in diameter). The expected detection limit for contrasted MRI is about 0.3 cm (2 × 2 pixels), better than noncontrasted MRI about 0.5 cm (3 × 4 pixels). Nevertheless, our results showed that no significant difference in cancer detection rates was found between contrasted and noncontrasted MRI (0.5% vs 0.4%, *p *= 0.24). It was assumed that the proportion of lost to follow-up and false-negative rate of contrasted MRI were similar to that of noncontrasted MRI because all examinations and follow-ups were performed in the same institution. Therefore, the possible explanation might be the same as previous comparison between MRI and LDCT. More subtle lung nodules do not necessarily bring out more lung cancers.

**Figure 3:**
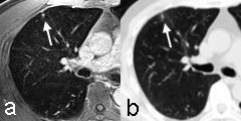
**Comparison of VIBE images from MRI and standard-dose CT image**. The (a) axial VIBE image and (b) standard-dose CT image showed a 0.3-cm subpleural nodule.

The prevailing argument that MRI is too time-consuming is no longer a valid concern. In this study, we demonstrated how a rapid lung MRI study can be performed with ease. Implementation of the parallel imaging technique has improved the image quality of HASTE in the axial and sagittal views. Currently, we acquire the VIBE sequence in axial, coronal, and sagittal planes by the reduction of TR/TE (2.9 ms/1.1 ms) and a factor of 2 with integrated parallel acquisition techniques (iPAT), which is allowed to cover the entire lung volume within a single breath hold. This optimization not only permits larger coverage but also reduces the pulsation artifact from the heart and large vessels. As shown in Figure 2, VIBE images can display clear branches of pulmonary vessels and bronchial tree with minimal pulsation artifacts. Although the resolution of contrasted MRI is still lower than that of standard-dose CT, MRI can show most nodules around 0.3 cm as standard-dose CT (Figure 3). The entire MRI examination of the lungs can be completed in less than 10 minutes, even if additional scanning is performed for suspicious nodules.

The radiation exposure should be justified when recommending the use of LDCT as a screening tool. Several authors have addressed the radiation hazards of multiple LDCT examinations [25-27]. When lung cancer screening involves the general population and is not targeted just at the high-risk population, the use of MRI can avoid the ethical problems of unnecessary ionizing radiation, which in the long term may contribute to lung cancer risk [28,29]. In addition, when contrast study is necessary, gadodiamide is noniodinated and has a better safety record with less nephrotoxicity [30].

In our results, there was no significance of cancer detection rate between smokers and never-smokers. However, most studies of lung cancer screening are aimed at smokers only. Many studies have demonstrated that lung cancer in never-smokers is an important public health issue, especially in females who tend to have more non-smoking associated cancers than males [31,32]. Clearly, to perform lung cancer screening in general population deserves more consideration and further researches.

Although the results of this study provide helpful insight into the use of MRI, the study has several limitations. A certain number of examinees were lost to follow-up. Moreover, follow-up periods and follow-up tools were not identical in all individuals. Standard-dose CT and plain chest radiography were performed only for those with suspected lung lesions; therefore, comparison of MRI with standard-dose CT and chest radiography for all nodules was not possible. Without a reference study, the percentage of false-negative cases could not be evaluated. In addition, like other one-arm observational nonrandomized trials, our study does not provide information on potential stage shift and reduction of lung cancer mortality by screening. Today, several randomized controlled trials of LDCT are under way worldwide [33-35]. We hope that our results will prompt subsequent randomized controlled trials of MRI that are not affected by lead time and length time bias and overdiagnosis.

## Conclusion

Based on the results of more than 10,000 MRI examinations, the 1.5T MRI with advanced pulse sequences approach described here, which takes less than 10 minutes to perform, is useful in lung cancer detection for asymptomatic individuals and avoids unnecessary ionizing radiation.

## Competing interests

The authors declare that they have no competing interests.

## Authors' contributions

NYW, HCC, and JSK conceived of the study concept and participated in its design. HCC, JSK, YCC, PWL, and WCL carried out imaging data analysis. NYW performed literature research, data collection, statistical analysis, and manuscript drafting and editing. HCC and DML helped to edit the manuscript. CYC participated in clinical study and coordination. All authors read and approved the final manuscript.

## Pre-publication history

The pre-publication history for this paper can be accessed here:

http://www.biomedcentral.com/1471-2407/11/242/prepub
